# Climate change induced atmospheric iodine enrichment – a paradoxically beneficial contribution

**DOI:** 10.1016/j.advnut.2025.100437

**Published:** 2025-04-29

**Authors:** Peter PA Smyth, Colin D O’Dowd

**Affiliations:** 1 From the UCD School of Medicine, University College Dublin, Dublin, Ireland; 2 Ryan Institute’s Centre for Climate & Air Pollution Studies, School of Physics, University of Galway, Galway, Ireland

Dear Editor:

In their recent Perspective, Liang et al. [1] used machine learning models XGPost clarified by SHapley Additive exPlanations to examine changes in the prevalence of iodine deficiency disorders over the period 1990–2021 with predictions extended to 2050. Despite evidence of improvements in global iodine deficiency disorder status, this unique study identified the need for continuing vigilance in at risk populations, particularly recommending the continuation of universal salt iodization. Although the study extended predictions to 2050, we would postulate that looking into the distant future, global iodine bioavailability may, as a result of global warming arising from climate change, show a steady increase [2]. Global warming has induced a reduction of the polar ice caps, together with manmade greenhouse gas pollution, and has been shown to generate volatile iodines [3]. The major factor driving the release of volatile iodines from seawater is the reaction of inorganic iodide, the major iodine speciation in the upper levels of seawater, with ozone (O3) generated as a pollutant in the lower atmosphere. The reaction shown below produces volatile iodines I_2_ and HOI.

O_3_ + H+ + I^–^ > HOI + O_2_

H+ + HOI + I^–^ > I_2_ + H_2_O

The progressive increase in such iodine has resulted in a 3-fold increase in iodine content of ice cores [4,5]. Analysis of this data has predicted that in the near future, we may see the highest global iodine concentrations in the past 127,000 y [6]. Although not an imminent prospect, increased atmospheric iodine may have an extended lifespan as it has been postulated that the current level of greenhouse gasses may extend the current interglacial period from an expected 10,000 y cycle to a potential 500,000 y [7]. Human fossil fuel use may, therefore, create a superinterglacial period that has overridden expected glacial cycles [7].

Although such iodine concentrations are never likely to reach pathological levels, the Japanese experience where daily dietary iodine intakes of ∼1 mg, orders of magnitude greater than western intake, has shown that high iodine concentrations can be readily tolerated [8], suggesting that the modest increases projected from climate change would be largely beneficial. Gaseous iodine released in the atmosphere can be returned to Earth via rainfall, where it enriches soil and, therefore, foodstuffs. The possibility of a contribution to iodine intake of gaseous iodines imbibed through respiration has been demonstrated in our own work [9], and the wider question of fresh air contributing to human nutrition (aeronutrients) has recently been explored [10]. Higher intakes, such as those recorded by populations living adjacent to a seaweed-abundant area (seaweed hotspot), might indicate levels that could be achieved during a prolonged period of global warming. Highest levels over the seaweed hotspot were equivalent to a supplementary intake of 46–81 ug/d (WHO recommended daily adult intake of 120 μg) [2,9]. The concentrations of atmospheric iodine likely to be achieved in the immediate future would not significantly affect population health but have the potential over a longer period of improving universal human health, particularly cognitive abilities in at risk populations [1,2].

Although iodine increases arising from global warming will not alter the predicted trends in the time frame covered by Liang et al. [1], they may serve as an additional contribution to understanding future global nutritional requirements. Should mankind survive the feared consequences of climate change, the current and probably future increase in global iodine status and associated dietary intake may have a paradoxically beneficial effect on human development and future evolutionary prospects.

## Funding

The authors report no funding received for this study.

## Conflict of interest

The authors report no conflicting interests.

## References

[1] Liang D., Wang L., Zhong P., Lin J., Chen L., Chen Q. (2025). Perspective: global burden of iodine deficiency: insights and projections to 2050 using XGBoost and SHAP. Adv. Nutr..

[2] Smyth P.P., O'Dowd C.D. (2024). Climate changes affecting global iodine status. Eur. Thyroid J..

[3] Carpenter L.J., Chance R.J., Sherwen T., Adams T.J., Ball S.M., Evans M.J. (2021). Marine iodine emissions in a changing world. Proc. Math. Phys. Eng. Soc..

[4] Legrand M., McConnell J.R., Preunkert S., Arienzo M., Chellman N., Gleason K. (2018). Alpine ice evidence of a three-fold increase in atmospheric iodine deposition since 1950 in Europe due to increasing oceanic emissions. Proc. Nat. Acad. Sci. U S A..

[5] Cuevas C.A., Maffezzoli N., Corella J.P., Spolaor A., Vallelonga P., Kjær H.A. (2018). Rapid increase in atmospheric iodine levels in the North Atlantic since the mid-20th century. Nat. Commun..

[6] Corella J.P., Maffezzoli N., Spolaor A., Vallelonga P., Cuevas C.A., Scoto F. (2022). Climate changes modulated the history of Arctic iodine during the last glacial cycle. Nat. Commun..

[7] Maslin M.T. (2020). Tying celestial mechanics to Earth’s ice ages. Phys. Today.

[8] Nagataki S. (2008). The average of dietary iodine intake due to the ingestion of seaweeds is 1.2 mg/day in Japan. Thyroid.

[9] Smyth P.P.A., Burns R., Huang R.J., Hoffman T., Mullan K., Graham U. (2011). Does iodine gas released from seaweed contribute to dietary iodine intake?. Environ. Geochem. Health.

[10] Fayet-Moore F., Robinson S.R. (2024). A breath of fresh air: perspectives on inhaled nutrients and bacteria to improve human health. Adv. Nutr..

